# VSD Surgical Closure in Colombia in Children with Secondary Pulmonary Hypertension. Does Altitude Influence Postoperative Pulmonary Pressure?

**DOI:** 10.1007/s00246-024-03697-1

**Published:** 2024-11-15

**Authors:** Linibeth Cruz-Baquero, Nicolas Molano-Gonzalez, Daniel García-Vargas, Alberto García Torres

**Affiliations:** 1 Interventional Pediatric Cardiologist Congenital Heart Institute, Fundación Cardioinfantil-Instituto de Cardiología, Bogotá, Colombia; 2 Clinical Research Group, School of Medicine and Health Sciences, Universidad del Rosario, Bogotá, Colombia; 3 General Physician. Fundación Cardioinfantil-Instituto de Cardiología, Bogotá, Colombia; 4 Chief of Pediatric Cardiology and Pediatric Interventional Cardiology. Congenital Heart Institute, Fundación Cardioinfantil-Instituto de Cardiología, Bogotá, Colombia

**Keywords:** Ventricular septal defect, Cardiac surgical procedures, Pulmonary hypertension, Altitude

## Abstract

A retrospective, cross-sectional, descriptive, observational study was carried out to describe the history of pulmonary hypertension in pediatric patients living at different altitudes following surgical correction of ventricular septal defect. Data from 40 patients who underwent surgery in La Fundacion Cardioinfantil was collected and used for our analysis. Bivariate analysis showed no significant relationship between altitude and pulmonary hypertension after ventricular septal defect closure. Unrelated to the main objective of our study, our investigation revealed that our population underwent surgical correction of VSD at older ages than expected. While previous publications demonstrate the benefit of intervention at 4 years of age or younger (19, 20), the average age in our studied population was found to be 7.8 years old. These patients had unfavorable hemodynamic parameters for ventricular septal defect closure, but our study showed that our patients benefited from surgery with an immediate satisfactory postoperative result. Patients transitioned from parameters indicating severe PH to mild PH within the first 24–48 h after surgery.

## Introduction

Up to 3–4% of patients with ventricular septal defects (VSD) continue to develop pulmonary hypertension (PH) after surgical closure of the defect (1, 2). A frequently considered aggravating prognostic factor for patients with congenital heart defects is the presence of hypoxemia in patients living at altitudes of 1500 m above sea level or higher (3, 4, 5, 6). This investigation sought to describe the impact of living at these altitudes in the evolution of postoperative PH secondary to VSD after surgical closure in La Fundacion Cardioinfantil. (7) To our knowledge, no articles have previously explored this relationship.

## Materials and Methods

### Study Design

A retrospective, cross-sectional, descriptive, observational study was carried out to describe the history of PH in pediatric patients following surgical correction of VSD. (2, 8).

Demographic characteristics described in our study included: age in months, sex, city of permanent residency and altitude of permanent residency.

Presurgical hemodynamic data measured by two-dimensional echocardiography 24 h before cardiac catheterization included only one parameter: pulmonary artery systolic pressure (PASP). Further presurgical data obtained by catheterization was: pulmonary artery mean pressure (PAmP), pulmonary vascular resistance (PVR), systemic vascular resistance (SVR), PVR/SVR index, PASP, systolic aortic pressure (SAoP), diastolic aortic pressure (DAoP), baseline QP/QS, SVR after O2 administration, PVR after O2 administration, PVR/SVR index after O2 administration, QP/QS after O2 administration and QP/QS after Nitric Oxide administration. This last parameter had to be removed from the study, given that very few patients in our studied population received NO and therefore data was considered insufficient for statistical analysis.

The associated comorbidities of the studied population were categorized into 4 groups: metabolic, pulmonary, genetic and patients with no associated comorbidities. The size of the VSD measured by transthoracic Doppler echocardiography was classified using parameters such as those used by Noe Mohammad et, al (9), classifying defects as small (< 3 mm), medium (3–6 mm) and large (> 6 mm). Location of the defect described with echocardiography was labeled as either perimembranous, inflow tract, outflow tract, muscular or subarterial.

Postoperative data used in our study included PASP measured by echocardiography in the first 24—48 h after the immediate postoperative period. The administration or not of vasodilator drugs (oxygen, sildenafil and bosentan) (10, 11) in the postoperative period was also taken into account.

### Study Population

Patients included in our study covered all children who had an isolated VSD (with no other associated cardiac defects) and secondary PH, who underwent surgical closure at La Fundación Cardioinfantil during a 6 year period from January 2017 up to April 2023. (12, 13, 14).

Exclusion criteria included children with no PH and children with other associated cardiac defects at the time of cardiovascular surgery; in other words, children who did not have VSD as their main diagnosis. (13, 14).

Data for city and altitude of permanent residency was collected for each of our patients. Only one adult was included in our study.

### Data Analysis

Qualitative variables were reported with absolute and relative frequencies while quantitative variables were reported with either arithmetic means and standard deviations or median and interquartile ranges, depending on the normality of the variable evaluated through the Shapiro–Wilk test.

To evaluate the statistical associations between RVSP and clinical and demographic variables, a bivariate analysis was conducted. Mean differences and the Spearman correlation coefficient were calculated, along with their corresponding p-values. All statistical analysis were performed using R software version 4.2.1.

## Results

52 patients were selected using the inclusion criteria of our study, of which 12 were excluded since they did not have enough data for complete data analysis. Of the 40 patients studied, 52% were male and the mean age was 7.8 years old (sd 7.14).

Univariate analysis of the presurgical echocardiographic data found PASP to be 67.06 mmHg (sd 16.61). Analysis of catheterization data described the following variables: mean PAmP 46.4 mmHg (sd 19.22 mmHg), mean PVR 6.8926 WU (sd 6.67 WU), mean SVR 26.80 WU (sd 17.54 WU), mean PVR/SVR of 0.27 (sd 0.23), mean PASP 62.84 mmHg (sd 21.50 mmHg), PADP 27.63 mmHg (sd 16.51 mmHg), mean SAoP 89.33 mmHg (sd 17.61 mmHg) and DAoP 52.79 mmHg (sd 14.40 mmHg). Mean baseline QP/QS was 2.81 (sd 2.04). Variables after O2 administration were as follows: mean PVR was 4.40 WU (sd 3.80 WU), mean SVR was 23.86 WU (sd 13.14 WU), mean PVR/SVR was 0.18 (sd 0.11), and mean QP/QS was 4.06 (sd 3.22).

On the other hand, mean immediate postoperative measurements of PASP using Doppler echocardiography was 43.12 mmHg (sd 22.52 mmHg). During this period 75% of patients required pharmacological management while 25% did not. Mean duration of pharmacological treatment was 28.6 days (sd 31.18 days). Patient classification into the different comorbidity groups mentioned previously revealed 45% of our studied population had no associated comorbidity, 42% had genetic comorbidities, 10% had metabolic comorbidities and 2.5% pulmonary comorbidities. A not statistically significant association was found between pulmonary comorbidities and high altitude. However, no other comorbidities showed any statistically significant associations with the other variables analyzed.

VSD location was as follows: 57% had a perimembranous VSD, 20% had inflow tract VSD, 15% subarterial VSD, 5% muscular VSD, and 2.5% had outflow tract VSD. As for the size of the defect, 95% of our study population had a large VSD, 5% had a medium VSD and there were no small VSDs. Table 1

**Table 1:** Demographic and clinical variables. mean and standard deviation or absolute and relative frequencies are reported

Variable	*n* = 40	
Sex, male	21	52,50%
Age (years)	7,87	± 7,14
Altitude	1563,45	± 1.195,85
Comorbidities		
Genetic	17	42,50%
Metabolic	4	10,00%
Pulmonary	1	25,00%
No associated comorbidities	18	45,00%
VSD Location		
Muscular	2	50,00%
Perimembranous	23	57,50%
Subarterial	6	15,00%
Inflow tract	8	20,00%
Outflow tract	1	25,00%
VSD size		
Large	38	95,00%
Medium	2	5,00%
Pharmacological treatment		
None	13	32,50%
User	27	67,50%
Presurgical data		
Echocardiogram		
PASP	67,06	± 16,61
Catheterization		
PAmP	46,4	± 19.22
PVR	6,89	± 6.67
SVR	26,8	± 17.55
PVR/SVR	0,27	± 0.23
PASP	62,84	± 21.50
PADP	27,63	± 16.51
SAoP	89,33	± 17.61
DAoP	52,79	± 14.40
B-QP/QS	2,81	± 2.04
O2 QP/QS	4,06	± 3.22
O2 PVR	4,4	± 3.80
O2 SVR	23,86	± 13.14
O2 PVR/SVR	0,18	± 0.11
TT	29,12	± 30.70
Postsurgical data		
Echocardiogram		
PASP	43,12	± 22.52

The average altitude of permanent residency was 1563.45 m (sd 1195.85) (this data was obtained based on the city of permanent residency). Bivariate analysis found that patients with associated pulmonary comorbidities lived at high altitude (2640 m above sea level). VSDs with muscular and outflow tract location were also found in patients living in high altitude (2640 m above sea level). Patients with large VSDs lived at moderate altitudes, at an average of 1628 m above sea level (sd 1191.53), while medium-sized VSDs lived in low altitudes, on average around 333 m above sea level. None of the previously mentioned associations with altitude were found to be statistically significant. (4, 5, 6).

Table 2 describes other associations found in our bivariate analysis. No statistically significant relations were found between the PASP measured by echocardiography 24–48 h after surgery and the variables: gender, comorbidities, size and location of the VSD.

**Table 2:** Bivariate Analysis of RV Systolic Pressure Measured by Echo with Categorical Variables. The effect measure is the mean difference, with the first category assumed as the reference category; only its mean is reported without a p-value. Confidence intervals are reported in parentheses

		Effect measure	p-value
Gender	Female	64,125	–
	Male	5.875 (− 6.122, 17.872)	0,32,525,115
Comorbidities	Genetic	64,6	–
	Metabolic	9.9 (− 9.819, 29,619)	0,31,256,866
	Pulmonary	− 3.6 (− 39.793, 32.592)	0,84,001,722
	No associated comorbidities	3.567 (− 37.792, 32.592)	0,59,460,981
VSD Location	Muscular	60	–
	Perimembranous	3.882 (− 19.969, 27.734)	0,74,097,663
	Subarterial	9.4 (− 17.295, 36.095)	0,10,883,509
	Inflow tract	19 (− 6.582, 44.582)	0,10,883,509
	Outflow tract	− 20 (-59,078, 19,079	0,10,883,509
VSD SIZE	Large	67,29	–
	Medium	− 7.29 (− 42.23, 27.649)	0,67,305,617
PHARMACOLOGICAL TREATMENT	None	60,667	–
	User	8.899 (− 4.251, 22.048)	0,17,717,502

Within the bivariate analysis shown in Table 3, an statistically significant relation (p 0.026) was reported between PASP measured through catheterization and postoperative PASP measured echocardiographically.

**Table 3:** Bivariate Analysis of RVSP by Echo with Quantitative Variables. The Spearman correlation coefficient and p-value are provided

	correlation	p-value
Age (years)	− 0.014 (− 0.3161, 0.336)	0,937,453,552
Altitude (mts)	− 0.232 (− 0.538, 0.127)	0,200,500,897
RVSP	0.428 (0.093, 0.676)	0,014665602
PVR	− 0.173 (− 0.492, 0.187)	0,343,673,412
SVR	− 0.091 (− 0.431, 0.272)	0,626,854,083
PVR/SVR	− 0.12 (− 0.455, 0.245)	0,519,404,413
PASP	0.404 (0.052, 0.667)	0,026703825
PADP	0.315 − 0.051, 0.607)	0,089534068
SAoP	− 0.008 (− 0.361, 0.347)	0,965,466,999
DAoP	− 0.07 (− 0.141, 0.291)	0,707,152,515
B-QP/QS	0.424 (0.088, 0.673)	0,015633925
O2 QP/QS	0.634 (0.18, 0.865)	0,011125735
O2 PVR	− 0.313 (− 0.711, 0.237)	0,255,352,872
O2 SVR	0.003 (− 0.51, 0.514)	0,99,162,076
O2 PVR/SVR	− 0.176 (− 0.176, − 0.631)	0,530,758,107
TT	− 0.812 (− 0.965, − 0.251)	0,014311793
PmAP	0.37 (.0.25, 0.637)	0,03692868

*PASP* pulmonary artery systolic pressure, *PAmP* pulmonary artery mean pressure, *PVR* pulmonary vascular resistance, *SVR* systemic vascular resistance, *PVR/SVR* resistance index, *PADP* pulmonary artery diastolic pressure, *SAoP* systolic aortic pressure, *DAoP* diastolic aortic pressure, *B-QP/QS* baseline QP/QS, *O2 QP/QS* QP/QS after O2 administration, *O2 PVR* PVR after O2 administration, *O2 SVR* SVR after O2 administration, *O2 PVR/SVR* resistance index after O2 administration. *TT* Postoperative treatment time, RVSP right ventricle systolic pressure by Echo

## Discussion

Our study was carried out in a high complexity, national and international referral center where adequate follow-up is impaired by shortcomings of our health system (7). Given the structural circumstances imposed, we were oftentimes forced to act quickly when the diagnosis is made, making a treat and repair strategy such as that established by Satoshi et. al. (15), unlikely in our social context, as it would entail a high risk for patient loss to follow-up and/or disease progression to group A—Eisenmenger, as detailed in the 2024 Task Force by Ivy D. et. al (16).

Mean presurgical data of our patients (Echocardiographic PASP of 67.06 mmHg–sd 16.61. Catheterization measured PAmP of 46.4 mmHg, PASP of 62.84 mmHg and SAoP of 89.33 mmHg) indicated severe pulmonary pressure values placed at around 70.34% of systemic pressure values. (17, 18) Mean PVR/SVR index was 0.27 (sd 0.23) and mean PVR was 6.89 WU (sd 6.67). Oxygen administration revealed a significant average decrease in the PVR/SVR index of 0.18 along with a doubling in the QP/QS compared to baseline (an expected increase, due to larger blood flow in the shunt caused by the drop in pulmonary vascular resistance.) Comparing preoperative PASP measured by catheterization against the PASP measured by echocardiography in the first 24–48 h after surgery, a mean reduction of 43.12 mmHg was observed. The previous reduction conveys a transition from severe PH to mild PH (19).

Our investigation revealed that our population underwent surgical correction of VSD at older ages than expected. While previous publications demonstrate the benefit of intervention at 4 years of age or younger (20, 21, 22, 23, 24), the average age in our studied population was found to be 7.8 years old. We believe our work provides an unexpected result, as our experience impacts current practices, given that our population lies outside of the surgical candidate recommendations provided by the 2024 task force (16, 25). Our patients had a post-tricuspid shunt, with RVP > 6 UW-m2, which would place them within the “gray area” established in tables No. 7—8 of the publication by Ivy et al. Other studies that support this claim include the article published in 2022 by Lotto et. al. (21, 22, 23, 26, 27), which mentions the great challenge of defect closure in patients with pulmonary hypertension. This study establishes that patients with PVR > 6 WU, along with other parameters should preferably receive initial pharmacological treatment before undergoing surgery, given the high risk and poor prognosis for these patients. Along the same line we can find the editorial comment carried out by Ivy D. and Frank B. (28) which categorizes patients with a PVR between 4 and 8 WU/m2, as borderline candidates for defect closure. (22, 26, 27).

Few literature can be found describing PH evolution following VSD closure in children. (24, 25). Our review of existing literature found a study published in 1968 by Lillehei CW, et al., in which the evolution of 57 children who underwent surgical closure was described, some being under 2 years old when surgery was performed. Presurgical catheterization data reported high PVR and poor postoperative results were mentioned, highlighting a high mortality rate in the postoperative period (29, 30).

Some of our study’s findings correlate with previous literature, such as the distribution of VSD location. Perimembranous VSD is the most common, followed by inflow tract VSD and to a lesser extent muscular and outflow tract VSD (9).

We documented a statistically significant association (p < 0.005) between preoperative PASP measured echocardiographically and PAmP measured by catheterization (17, 31). A positive association was also detected between preoperative and postoperative PASP measured echocardiographically and both PASP and PADP measured by catheterization (31). Both of these associations support the practice of echocardiographic monitoring of PASP, as it yields a very reliable result and it has a strong and positive relationship with PAmP measured by catheterization. (17) When reviewing previous literature we found that PASP measured by echocardiography holds a strong correlation with PASP measured by catheterization (32, 33, 34, 35, 17), although it has not yet been possible to establish whether this is the most accurate parameter for noninvasive PH assessment.

The lack of data in pediatric population presents a considerable challenge for pediatric cardiologists and cardiac surgeons alike. Previous publications such as that carried out by Ivy D. et. al (16). establishes criteria for closure of defects such as patent ductus arteriosus and atrial septal defect with PH, but without guidelines for VSD. Currently there are no clear protocols for electing surgical candidates in children with VSD and PH. Along with this, we are also confronted with the uncertainty of the prognosis and long-term behavior of these patients. (8, 36).

Colombia offers a unique opportunity due to its heterogeneous population living in different altitudes, (4, 5, 6) however our study was unable to demonstrate any statistically significant associations between altitude of permanent residency and any other variable, including postoperative PH. We hope that by addressing the limitations described previously, a longer postoperative follow-up can be established, allowing us to further ascertain whether a relationship exists. (37).

## Conclusions

Despite borderline unfavorable parameters for surgical closure of VSD (PVR > 6 WU, PVR/SVR 0.27) and no previous administration of pulmonary vasodilators, our patients benefited from surgery with an immediate satisfactory postoperative result. Patients transitioned from parameters indicating severe PH to mild PH within the first 24–48 h after surgery.(36).

## Limitations

We are unable to know whether mild PH present in the postoperative period in our patients is attributable to the late age of the patients or due to altitude, given that the majority of our patients reside at least at moderate altitude and the reference center where surgery took place is located at high altitude (Bogotá—2625 m above sea level). Follow-up studies could address this by describing the relationship found when surgical interventions take place at sea level.

Despite our heterogeneous population residing at a great range of altitudes, no statistically significant relationship was found in this study, as such the existence of a relationship can’t be either ascertained or ruled out by our study.

Another limitation in our study lies in the number of patients included, with a larger sample a more accurate relation between pulmonary hypertension after VSD surgical closure could be seen. Future studies should involve other centers, allowing for a larger cohort to be analyzed and therefore for a better estimate to be retrieved. Future studies could also focus on ascertaining a relationship between different comorbidities and possibility for surgery in patients with VSD with PH, as these could also prove key to excluding candidates for surgery.

## Data Availability

Data is available in our databases.
